# Draft Genome Sequences of Two *Staphylococcus aureus* Strains Isolated in Succession from a Case of Bacteremia

**DOI:** 10.1128/genomeA.00259-17

**Published:** 2017-06-08

**Authors:** M. D. S. Basco, J. R. Revollo, P. B. McKinzie, S. Agnihothram, A. Kothari, M. Saccente, M. E. Hart

**Affiliations:** a Division of Microbiology, National Center for Toxicological Research, U.S. Food and Drug Administration, Jefferson, Arkansas, USA; b Division of Molecular and Genetic Toxicology, NCTR, U.S. Food and Drug Administration, Jefferson, Arkansas, USA; c Department of Internal Medicine, University of Arkansas for Medical Sciences, Little Rock, Arkansas, USA; d Department of Microbiology and Immunology, University of Arkansas for Medical Sciences, Little Rock, Arkansas, USA

## Abstract

*Staphylococcus aureus* strains MEH1 and MEH7 were successively isolated from the blood of a patient with recurrent bacteremia. The submitted draft genomes of strains MEH1 and MEH7 are 2,914,972 and 2,911,704 bp, respectively.

## GENOME ANNOUNCEMENT

*Staphylococcus aureus* is a leading cause of nosocomial bacteremia that often leads to life-threatening diseases, such as osteomyelitis and infective endocarditis (1–7). *S. aureus* strains MEH1 and MEH7 were isolated 7 days apart from the blood of a bacteremic patient who had mitral and aortic valve endocarditis, L4-5 spinal diskitis, and osteomyelitis. These strains were sequenced to study the factors contributing to the persistence of the infection during the course of antibiotic treatment.

Bacterial cell pellets were suspended in Tris-EDTA buffer (560 µL) with lysostaphin (1 µg/mL) and RNase (10 µg/mL) and incubated for 30 min at 37°C. Cell suspensions were further incubated with 30 µL of 10% SDS and 10 µL of proteinase K (20 mg/mL) (Thermo Fisher Scientific, Waltham, MA, USA) for 30 min. Five molar NaCl (100 µL) was added, vortexed before the addition of 80 µL of cetyltrimethylammonium bromide (10% wt/vol)–NaCl solution (4.1% wt/vol) (Sigma-Aldrich, St. Louis, MO, USA), and incubated at 65°C (10 min). To this, 700 µL of chloroform/isoamyl alcohol (24:1) (Sigma-Aldrich) was added and centrifuged at 9,400 × *g* for 5 min. To the upper aqueous layer an equal volume of phenol-chloroform/isoamyl alcohol (25:24:1, pH 6.7) (Sigma-Aldrich) was added and centrifuged for 10 min. DNA from the upper layer was precipitated with isopropanol at a ratio of 0.6:1. The DNA pellet obtained was washed with 70% ethanol, air-dried, and suspended in water.

Genomic DNA libraries were independently dual-indexed (8 × 2) with the Nextera DNA library prep kit (Illumina, San Diego, CA, USA) as recommended by the manufacturer. Libraries were pooled and purified with a GeneRead size-selection kit (Qiagen, Valencia, CA, USA); the pooled DNA libraries were quantified with a Qubit dsDNA high-sensitivity assay kit (Thermo, Fisher Scientific) and analyzed with a high-sensitivity DNA chip (Agilent, Santa Clara, CA, USA). Libraries were paired-end (76 × 2) sequenced in a single dual-indexed (8 × 2) paired-end (76 × 2) NextSeq500 using a Mid Output (150 cycle) version 2 kit (Illumina) as recommended by the manufacturer. The resulting sequences were assembled into contiguous (contigs) fragments by the SPAdes version 3.6.2 genome assembler for Galaxy Tool version 1.2 with default parameters. The MEH1 and MEH7 draft genomes were determined to be 2,914,972 and 2,911,704 bp in length, distributed as 66 and 75 contigs, and based on average *de novo* assembly coverages of 110× and 62×, respectively. The G+C content was 32.7% for both strains. The genome of MEH1 contains a total of 3,123 genes that include 2,977 coding genes, 4 rRNAs (5S, 16S, and 23S), 56 tRNAs, and 82 pseudogenes. The genome of MEH7 contains a total of 3,142 genes that include 2,987 coding genes, 3 rRNAs (5S, 16S, and 23S), 65 tRNAs, and 83 pseudogenes. Annotations were performed using the NCBI Prokaryotic Genome Annotation Pipeline (8).

### Accession number(s).

The genome sequences of *S. aureus* strains MEH1 and MEH7 have been deposited at DDBJ/EMBL/GenBank with the accession numbers MUIR00000000 and MUIS00000000, respectively. The versions described here are MUIR00000000.1 and MUIS00000000.1 for strains MEH1 and MEH7, respectively.
